# Early 24-Hour Changes in Systemic Immune–Inflammation Index Predict Acute Kidney Injury and Mortality in ICU Patients

**DOI:** 10.1155/emmi/4949299

**Published:** 2025-08-19

**Authors:** Fei Gao, Lan Yang, Yizhe Chen, Hongyang Xu, Ting Yang

**Affiliations:** Department of Critical Care Medicine, The Affiliated Wuxi People's Hospital of Nanjing Medical University, Wuxi People's Hospital, Wuxi Medical Center, Nanjing Medical University, Nanjing, China

**Keywords:** acute kidney injury, critically ill patients, mortality, systemic immune–inflammation index

## Abstract

**Background:** To determine whether early dynamic changes in the systemic immune–inflammation index (SII) improve prediction of acute kidney injury (AKI) and 1-year mortality in critically ill patients.

**Methods:** In this retrospective cohort study of 17,491 ICU admissions from the MIMIC-IV database, we calculated three SII metrics within the first 24 h of ICU stay: the 24-h SII_slope and the extreme values (SII_min, SII_max). LASSO-selected multivariable logistic regression was used to predict AKI, and Cox proportional hazards models assessed associations with 1-year mortality. A prognostic nomogram integrating SOFA score, APS III score, and log-transformed SII_min and SII_max was developed using the rms package in R. Model performance was evaluated by AUC of ROC curves, calibration plots, decision curve analysis (DCA), and Kaplan–Meier survival curves stratified by SII quartiles.

**Results:** The LASSO-based logistic model identified a steeper 24-h SII_slope as an independent predictor of AKI (AUC 0.739; patients who developed AKI had significantly higher predicted risk than those who did not). Higher SII_min and SII_max were each associated with reduced 1-year survival (log-rank *p*=0.047 for SII_min quartiles). The nomogram for 1-year mortality demonstrated excellent discrimination (AUC 0.823) and good calibration, and DCA confirmed its clinical utility.

**Conclusions:** Early dynamic changes in SII—especially the 24-h slope—and the first-day SII extremes independently predict AKI and long-term mortality in ICU patients. A nomogram combining SII metrics with standard severity scores may facilitate individualized risk stratification in critical care.

## 1. Introduction

Critically ill patients often experience a dysregulated inflammatory and immune response, which contributes significantly to organ dysfunction and mortality. Conventional severity scoring systems, such as the SOFA and the APS III, provide overall estimates of illness severity but may not fully capture the dynamic immune status of patients during their ICU stay [1]. Recently, dynamic inflammatory markers like the neutrophil-to-lymphocyte ratio (NLR) and the platelet-to-lymphocyte ratio (PLR) have shown prognostic utility in sepsis and other critical conditions [2, 3]. More notably, the systemic immune–inflammation index (SII), which integrates neutrophil, lymphocyte, and platelet counts, has been independently associated with mortality in various critically ill populations, including those with traumatic brain injury [4] and sepsis-associated acute kidney injury (AKI) [5]. These findings suggest that dynamic SII measurements may offer more nuanced prognostic insight than static severity scores alone, particularly by capturing shifts in inflammatory load and immune balance.

The SII is a composite biomarker. This index integrates three different blood cell parameters to represent the interplay between proinflammatory (neutrophils, platelets) and anti-inflammatory (lymphocytes) forces [6]. Elevated SII has been associated with adverse outcomes in various diseases, including cardiovascular and oncologic conditions [7]. However, previous studies have primarily focused on static SII values measured at a single time point (often on admission) [8], rather than changes in SII over time.

We note that the 24-h period represents a critical early phase of ICU care where initial inflammatory changes occur and interventions can be timely. We postulated that dynamic changes in SII during the first 24 h of ICU admission may offer additional prognostic information beyond any single measurement. In particular, the slope of SII over time could capture the trajectory of a patient's inflammatory response under critical illness, and extreme SII values (minimum and maximum within the first day) might reflect episodes of immune dysregulation. Therefore, the objective of this study was to evaluate the prognostic value of dynamic SII indices—including the 24-h SII_slope and first-day SII_min and SII_max—in predicting AKI and ICU patient survival. We further developed and validated a prognostic nomogram that integrates these SII-based biomarkers with conventional severity scores to facilitate individualized risk prediction for critically ill patients.

## 2. Methods

### 2.1. Study Cohort

Our study adheres to the STROBE statement and is a retrospective cohort study utilizing the MIMIC-IV database [9]. All methods were performed in accordance with the relevant guidelines and regulations. One author on our team (F.G.) has access to the database and is responsible for data extraction (certificate number: 48448818). Patients admitted to the ICU were eligible. We included patients who had the necessary laboratory data to calculate SII within the first 24 h of ICU admission (specifically, at least one measurement of neutrophil count, lymphocyte count, and platelet count). Patients with missing outcome data (e.g., unknown survival status) or implausible laboratory values were excluded. All data in MIMIC-IV are de-identified; thus, the requirement for informed consent was waived by the original data collectors.

Relevant baseline variables were collected for each patient, including demographics (age and sex), anthropometrics (weight, height, and body mass index), and comorbidities (e.g., heart failure, renal disease, liver disease, chronic obstructive pulmonary disease, coronary artery disease, history of stroke, and malignancy). We also extracted ICU interventions within the first 24 h (mechanical ventilation, vasopressor use, and sedative use) and initial severity of illness scores: SOFA score and APS III score (calculated upon ICU admission). Key vital signs (mean arterial pressure, heart rate, body temperature, and respiratory rate) and standard laboratory measurements during the first 24 h were recorded, including electrolytes, arterial blood gas values, and biomarkers such as lactate, BUN, creatinine, and troponin.

### 2.2. SII Calculation

For each patient, the SII was calculated as SII = (platelet count) × (neutrophil count)/(lymphocyte count). All counts were obtained from blood tests during the first 24 h of ICU admission. We captured the dynamic behavior of SII in two ways. First, we modeled the 24-h SII trend by performing a linear regression of SII values against time (in hours) over the initial 24-h window for each patient; the slope of this regression line was recorded as the SII_slope (per 24 h). This SII_slope_24 h represents the rate of change in the immune–inflammation index (a positive slope indicating increasing inflammation and a negative slope indicating a decreasing trend). We also extracted the minimum SII (SII_min) and maximum SII (SII_max) for each patient within the first 24 h. These extreme values reflect the lowest and highest immune–inflammatory states a patient experienced during the early critical period. If only one SII measurement was available on the first day, that value was considered as both SII_min and SII_max, and the slope could not be calculated (such patients were excluded from slope-based analyses). All SII metrics were calculated from the same set of laboratory data (complete blood counts) routinely collected as part of ICU care.

### 2.3. Outcomes

The primary outcome of interest was the development of AKI during the ICU stay. AKI was identified based on the KDIGO (Kidney Disease: Improving Global Outcomes) criteria [10], typically defined by changes in serum creatinine or urine output. In this study, patients were considered to have AKI if they met the KDIGO criteria for stage 1 or higher at any point during their ICU admission. The secondary outcome was long-term mortality, specifically all-cause mortality within 1 year after ICU admission. Survival time was calculated from the date of ICU admission to the date of death or last known follow-up (censored at 365 days for those surviving beyond 1 year). For patients who were discharged from the hospital, survival status was determined using follow-up records available in the database (which may include linkage to national death records for the follow-up period).

### 2.4. Statistical Analysis

We first constructed a multivariable logistic regression model to predict the odds of developing AKI during the ICU stay. To select the most predictive features for AKI, we employed LASSO regularization [11]. Candidate predictors included patients' demographic factors, baseline clinical variables, severity scores, and the SII metrics (SII_min, SII_max, and SII_slope). The LASSO method was applied with cross-validation to identify a subset of variables with nonzero coefficients, optimizing the model's balance between complexity and performance. The final logistic regression model was then fitted using these selected predictors. Model discrimination was evaluated by the area under the receiver operating characteristic curve (AUC) for AKI prediction. We also assessed model calibration by comparing predicted probabilities with observed incidence of AKI (via calibration plots).

To evaluate the association between SII metrics and long-term survival, we performed Cox proportional hazards regression analysis for 1-year mortality. In univariate analyses, we examined hazard ratios for mortality across quartiles of SII_min, SII_max, and SII_slope. We then built a multivariable Cox model including established risk factors and SII-based variables. Because SII_min and SII_max had skewed distributions, these were log-transformed (log10) before inclusion in the Cox model to meet model linearity assumptions. The proportional hazards assumption was checked for each covariate (and no significant violations were found). The primary Cox model included SOFA score, APS III score, log(SII_min), and log(SII_max) as covariates—based on clinical relevance and the results of the feature selection process for the mortality outcome.

Using the multivariable Cox model, we developed a prognostic nomogram to provide an individualized risk prediction for 1-year survival [12]. The nomogram was constructed with the aid of the rms package in R, mapping each predictor (SOFA, APS III, log SII_min, log SII_max) to a point scale and summing these to generate a total points score, which corresponds to a predicted probability of 1-year survival. The selection of these four predictors was guided by the model results (notably, SII_slope did not remain significant for long-term mortality in the presence of SII_min and SII_max, so it was not included in the final nomogram). We internally validated the nomogram performance using bootstrapped resampling (200 repetitions) to estimate optimism-corrected metrics.

The performance of predictive models was assessed on several fronts. Discrimination for binary outcomes was quantified by the AUC of the ROC curve. For survival models, we generated time-dependent ROC curves at the 1-year time horizon to calculate the AUC for 1-year mortality predictions [13]. Model calibration was evaluated through calibration curves plotting predicted versus observed 1-year survival probabilities; an ideal model would lie on the 45-degree line. We also performed decision curve analysis (DCA) to examine the clinical usefulness of the prediction models (both the AKI model and the mortality nomogram) [14]. DCA assesses the net benefit across a range of threshold probabilities, determining whether the model provides improvement in clinical decision-making compared to default strategies of treating all or no patients. Finally, Kaplan–Meier survival curves were constructed to visualize differences in survival over time stratified by quartiles of SII metrics. The log-rank test was used to compare survival distributions between groups. All statistical analyses were conducted using R software (Version 4.3.1) with appropriate packages for regression modeling and validation.

## 3. Results

### 3.1. Baseline Characteristics

**Figure 1:**
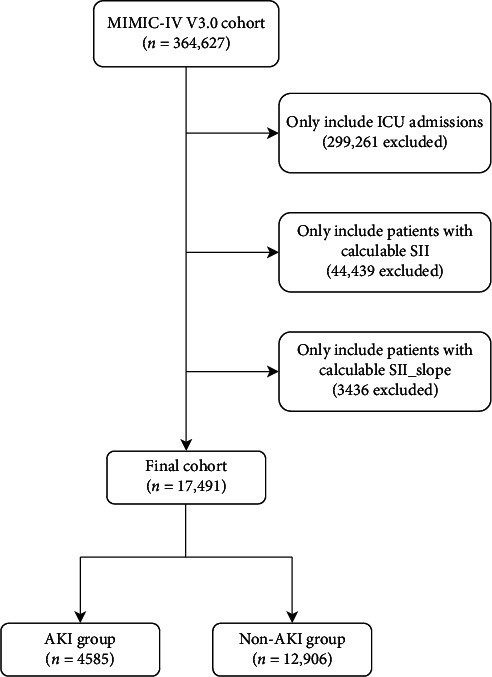
The study flowchart in this research illustrates the exclusion and inclusion criteria used to select the final cohort of 17,491 patients.

We selected the study cohort from the MIMIC-IV database. After excluding non-ICU hospital admissions, patients without any SII laboratory data, and the 3436 patients—about 16.4% of the cohort—who had only a single SII measurement in the first 24 h. Baseline characteristics of patients for whom the SII_slope could not be calculated are shown in Supporting Table 1. A final cohort of 17,491 ICU patients was identified. This cohort was divided into those who developed AKI during the ICU stay (AKI group, *n* = 4585) and those who did not (non-AKI group, *n* = 12,906), as shown in Figure 1. The baseline characteristics of the two groups are summarized in Table 1. Overall, AKI patients had higher severity of illness on admission, with a mean SOFA score of 6.19 versus 3.93 in non-AKI, and an APS III score 49.3 versus. 37.7 (*p* < 0.001). They also more frequently required critical interventions in the first 24 h, such as mechanical ventilation (52.7% vs. 26.8%) and vasopressors (49.0% vs. 29.6%, *p* < 0.001). Comorbid conditions like heart failure and chronic kidney disease were more prevalent in the AKI group. In laboratory results, with respect to immune-inflammation indices, there were notable differences: the AKI group had a higher average SII_max on the first day (2737 vs. 2247, *p* < 0.001) and a higher SII_min (2409 vs. 2066, *p* < 0.001) compared to non-AKI patients. Additionally, the mean 24-h SII_slope was positive in the AKI group (mean + 13.66), indicating rising SII, whereas it was lower in non-AKI patients (+7.54, *p* < 0.001). Thus, patients who developed AKI had a more pronounced inflammatory response early in their ICU course, as reflected by higher and increasing SII values.

### 3.2. AKI Prediction by Dynamic SII

**Figure 2:**
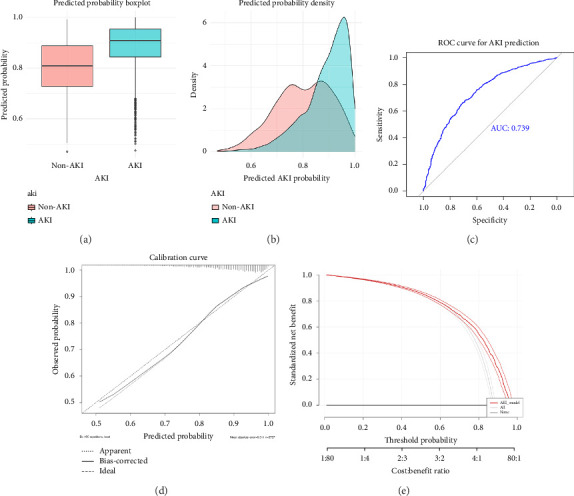
Performance of the multivariable logistic regression model for AKI prediction. (a) and (b) display the distribution of model-predicted AKI probabilities in patients who did and did not develop AKI. Patients who eventually developed AKI tended to have higher predicted risk scores at admission (median ∼0.80) compared to those who did not develop AKI (median ∼0.75), indicating the model's risk stratification aligns with actual outcomes. (c) Shows the receiver operating characteristic curve of the model, with an area under the curve (AUC) of 0.739 for discriminating AKI vs. non-AKI. (d) Calibration curve for AKI prediction model. Apparent (dotted line) and bias-corrected (solid line) calibration plots are shown, with the ideal 45° line (dashed) for reference. Model-predicted probabilities of acute kidney injury (AKI) are plotted on the *x*-axis against the observed AKI incidence on the *y*-axis. Bootstrap resampling (*B* = 100) was used to estimate optimism-corrected calibration. (e) Decision curve analysis of the AKI prediction model. Standardized net benefit (*y*-axis) is plotted across a range of threshold probabilities (*x*-axis) for three strategies: Using the logistic AKI model (red), treating all patients (“All,” thin red), and treating none (“None,” gray). The model confers positive net benefit over default strategies between threshold probabilities of approximately 0.3 and 0.8.

As shown in Figure 2, using the LASSO feature selection procedure on candidate variables, the key predictors of AKI that were identified included age, sex, SOFA score, APS III score, SII_max, and SII_slope. These six features were incorporated into the final logistic regression model for AKI. The model demonstrated fair discrimination with an AUC of 0.739 for predicting AKI (Figure 2(c)). Internal validation via cross-validation yielded a similar performance, suggesting the model was not overfit. The predicted risk probabilities for AKI were significantly higher in those who developed AKI than in those who did not (Figures 2(a) and 2(b)), reflecting good separation between the groups.

We examined the calibration of the AKI prediction model by comparing predicted and observed AKI incidence across deciles of risk. The calibration plot (Figure 2(d)) showed that the model's predictions were well aligned with observed outcomes, with most points lying near the ideal 45° line, especially in the mid-range of probabilities. This indicates that the model was neither systematically over-predicting nor under-predicting the risk of AKI. We also performed DCA to evaluate clinical utility (Figure 2(e)). The decision curve for the logistic model was above the net benefit of treating all patients or none across a broad range of threshold probabilities, which implies that using the SII-based model to guide interventions (for example, preventive strategies for AKI) would confer a net benefit to patients compared to default strategies. In summary, a dynamic increase in SII during the first ICU day (reflected by a steep SII_slope and high first-day SII_max) was an independent predictor of AKI, and incorporating SII metrics improved the ability to predict AKI risk beyond traditional scores.

### 3.3. SII and Long-Term Survival

We analyzed the relationship between early SII metrics and long-term survival outcomes. At the 1-year follow-up, the overall mortality in the cohort was approximately 20%. Kaplan–Meier survival analyses demonstrated that patients with more extreme or rising inflammatory indices had worse survival over time. In particular, individuals stratified to the highest quartile of SII_min or SII_max had a tendency toward lower 1-year survival compared to those in the lowest quartiles. Patients whose minimum SII in the first 24 h remained very high (indicating persistently elevated inflammation without a dip) experienced poorer survival rates than those whose SII dropped to lower levels early on. Those in the top quartile of SII_max (extremely high peak inflammation) also showed a trend toward higher mortality, though this difference did not reach statistical significance in our sample. Detailed Kaplan–Meier curves for survival stratified by SII_min and SII_max quartiles are provided in Supporting Figures 1 and 2. Notably, by the end of 1 year, the survival probability for the highest SII_min group was visibly lower than that of the lowest group (log-rank *p*=0.047), suggesting that even transient suppression of immune response (reflected in a low SII_min) might be protective. For SII_max, while the highest quartile had the lowest estimated survival, the differences among quartiles were not as pronounced (log-rank *p*=0.31), indicating considerable variability and potential confounding factors in that association.

**Figure 3:**
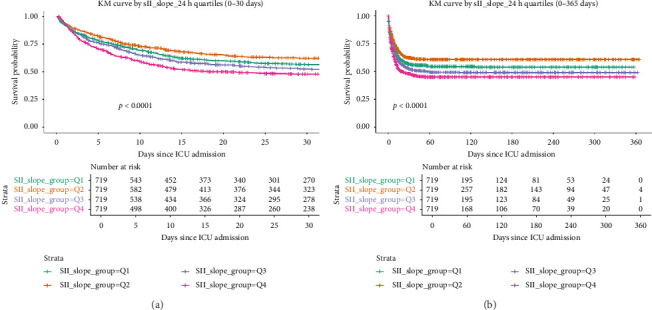
Kaplan–Meier survival curves stratified by the quartiles of 24-h SII_slope. (a) 30-day ICU survival for patients in quartiles Q1–Q4 of SII_slope. (b) 1-year survival for the same groups. Steeper positive SII_slopes (magenta line, Q4) correspond to rapidly increasing inflammation and are associated with substantially lower survival probabilities in both the short term and long term (*p* < 0.0001 for overall comparison). In contrast, patients in the lowest SII_slope quartile (green line, Q1), who had stable or decreasing SII levels, show the highest survival rates.

We also evaluated survival in relation to the SII_slope quartiles. Patients were categorized into four groups based on the quartile of their 24-h SII_slope (from the most negative slopes to the highest positive slopes).  Figure 3 illustrates the Kaplan–Meier survival curves for these groups over two time horizons: short term (30 days) and long term (365 days). Patients with the steepest positive SII_slope (Q4), meaning their inflammatory index rose rapidly in the first day, had significantly worse early survival than those with stable or decreasing SII (Q1 and Q2). As shown in Figure 3(a), over the first 30 days in the ICU, the highest slope group diverged early with much lower survival probability, while the lowest slope group (representing patients whose SII stayed constant or decreased) had the best short-term survival (log-rank *p* < 0.0001 for difference across groups). This trend persisted, albeit less dramatically, over the 1-year follow-up (Figure 3(b)): Patients in the highest SII_slope quartile continued to have the lowest cumulative survival, and those in the lowest quartile had the highest survival, with a statistically significant overall difference (*p* < 0.0001). These findings suggest that an increasing SII trajectory is an ominous sign associated with both acute and long-term mortality risk in critical illness. Conversely, patients whose SII response was flat or decreasing in the first 24 h (indicating better control of inflammation) fared better. Taken together, our results highlight that both static and dynamic SII indicators carry prognostic information: Even one-time extreme values of SII (very high or very low) have implications for mortality, and the direction of change of SII early in ICU admission is strongly associated with patient survival.

### 3.4. Nomogram for Mortality Risk

Based on the multivariate analysis of 1-year mortality, we constructed a nomogram that integrates key prognostic factors, including the SII-based indices. The final Cox proportional hazards model included SOFA score, APS III score, log(SII_min), and log(SII_max) as independent predictors of 1-year mortality, all of which were statistically significant. The model coefficients were translated into a nomogram to estimate an individual patient's probability of survival at 1 year.

**Figure 4:**
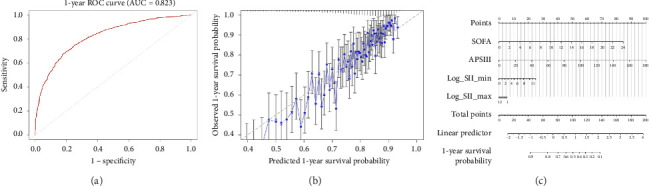
Nomogram and validation for 1-year mortality prediction. (a) The ROC curve for the Cox model's 1-year survival prediction (treated as a binary outcome), with an AUC of 0.823, indicating excellent discrimination. (b) The calibration plot for the nomogram, comparing predicted 1-year survival probabilities with observed outcomes; the blue line (with 95% confidence interval bars) closely follows the diagonal, demonstrating good agreement between predictions and actual survival. (c) On the right displays the nomogram, which assigns points to each predictor: SOFA score, APS III score (labeled “apsiii”), log_10 SII_min, and log_10 SII_max. By summing the points corresponding to a patient's values for each variable, one can determine the total points and map that to a predicted 1-year survival probability at the bottom scale.

In our cohort, the nomogram-predicted risk aligned well with observed mortality: The Hosmer–Lemeshow-type calibration test was non-significant, and visually the calibration curve (Figure 4(b)) showed only minor deviation from the perfect calibration line, mostly at the higher end of predicted risk. The concordance index (C-index) for the Cox model was 0.82, consistent with the ROC AUC of 0.823 at 1 year, reflecting a high ability to rank patients by risk. For example, the nomogram would predict that a patient with a SOFA of 10, an APS III of 100, an SII_min = 1500, and an SII_max = 8000 has around a 50% probability of 1-year survival, whereas a patient with more moderate values (SOFA 5, APS III 60, SII_min = 500, and SII_max = 2000) might have an estimated 80%–90% 1-year survival probability.

We also evaluated the clinical utility of the mortality prediction nomogram. DCA demonstrated a net benefit of using the nomogram to stratify mortality risk across a range of threshold probabilities (∼10%–60% 1-year mortality risk). In other words, interventions guided by the nomogram (such as intensified monitoring or early palliative care consult for those at highest risk) would likely result in better outcomes than a one-size-fits-all approach. The net benefit was especially higher than the “treat-all” or “treat-none” strategies in the 20%–40% risk threshold range, which is relevant for ICU decision-making.

Overall, the nomogram provides an interpretable tool to quantify long-term mortality risk at ICU admission by combining traditional severity scores with immune-inflammation markers. The strong performance of this model (Figure 4(a)) suggests that incorporating SII_min and SII_max (log-transformed) alongside SOFA and APS III meaningfully improves risk discrimination. For instance, SII_min and SII_max contributed roughly 20–30 points each on the nomogram scale for the ranges observed, indicating that a very high initial inflammatory burden can shift the prognosis considerably. This personalized risk prediction model could assist clinicians in identifying patients who are at high risk of death despite similar clinical scores, possibly due to extreme or unresolved inflammation.

## 4. Discussion

In this study, we comprehensively evaluated the prognostic significance of dynamic immune–inflammation indices during early ICU admission. We found that both static SII measures (the minimum and maximum values within 24 h) and dynamic changes in SII (24-h slope) are strongly associated with patient outcomes, specifically the risk of AKI and long-term mortality. To our knowledge, this is one of the first investigations to incorporate dynamic SII trajectories into critical care prognostication. Furthermore, we developed a combined prognostic model in the form of a nomogram that integrates SII-based biomarkers with conventional severity scores (SOFA and APS III), and we demonstrated that this model achieves excellent predictive performance for 1-year survival in ICU patients.

Our results indicate that a steeper increase in SII over the first day in the ICU is an independent predictor of AKI. Even after accounting for baseline illness severity and other risk factors, the SII_slope remained a significant contributor to AKI risk. This finding aligns with the concept that persistent or rapidly escalating inflammation can precipitate organ dysfunction. AKI in critical illness often has a multifactorial etiology, but systemic inflammation and immune cell activation are known to play important roles in microvascular damage and tubular injury [15]. A rising SII may reflect increasing neutrophil counts (and neutrophil activation) coupled with relative lymphopenia, as well as thrombocytosis or platelet activation—all of which can contribute to a proinflammatory, prothrombotic state in the renal microcirculation [16]. Thus, the SII_slope could serve as an early warning signal for clinicians: Patients whose inflammatory index is climbing quickly might benefit from closer monitoring of renal function or early interventions to mitigate kidney stress (such as optimizing hemodynamics or avoiding nephrotoxins). Notably, our logistic model showed improved discrimination for AKI when adding SII_max and SII_slope to traditional predictors (AUC 0.739), and it was well-calibrated to observed AKI incidence. This highlights the potential utility of dynamic inflammation markers as adjuncts to existing AKI prediction tools.

Moreover, the combination of a high SII_max in the first 24 h with standard clinical variables enhanced AKI risk stratification. We observed that patients who reached very high SII values shortly after ICU admission had markedly higher odds of developing AKI. This may capture the impact of acute surges of inflammation (for example, a cytokine storm or neutrophil extracellular trap formation) that can acutely impair organ perfusion or directly injure tissues [17, 18]. From a practical standpoint, SII is easily computed from routine complete blood counts, and trending this value could be incorporated into ICU electronic health records to flag patients with accelerating inflammation.

Interestingly, we found that extreme early SII values carry prognostic implications for long-term survival. Patients whose SII remained elevated throughout the first day (high SII_min) had worse outcomes at 1 year. In contrast, if a patient's SII dropped to a very low level at some point (low SII_min), it might indicate a more effective regulation or resolution of the initial inflammatory response, which was associated with better survival. On the other hand, patients experiencing an extremely high peak in SII (high SII_max) tended to have poorer survival, although in our cohort this association did not reach statistical significance after adjustment. These findings support the notion that both an overwhelming inflammatory response and the inability to suppress inflammation are detrimental [19, 20]. A transient but excessive immune activation (reflected by high SII_max) might lead to irreversible damage early, whereas a failure to ever mount a lower inflammatory state (reflected by high SII_min) could indicate ongoing injury or secondary complications.

Our survival analysis with SII_slope quartiles further reinforced that the trajectory of immune response is crucial. A persistently rising or unremitting inflammatory trajectory (highest SII_slope group) was associated with significantly higher mortality, even when initial severity scores were considered. This dynamic perspective complements static biomarkers: For example, two patients might both have a moderately high SII on admission, but if one's SII is climbing and the other's is decreasing, our data suggest their prognoses could diverge. Prior studies have linked related markers such as the NLR and platelet counts to outcomes in critical illness and sepsis [21–23]. SII, by integrating neutrophils, lymphocytes, and platelets into a single composite index, may better capture this complex immune balance. Our findings extend these observations by demonstrating that not only the magnitude but also the time course of SII matters in predicting outcomes.

We developed a four-variable nomogram that blends SOFA, APS III, and two early inflammatory metrics (SII_min and SII_max) to predict 1-year mortality. The model discriminates well (AUC = 0.823) and calibrates closely to the 45-degree line, while decision-curve analysis confirms a consistent net clinical benefit. To illustrate its use, suppose a patient's first-day data are SOFA 9, APS III 85, SII_min 5.6 × 10^3^, and SII_max 9.8 × 10^3^; the nomogram yields ≈55% predicted 1-year mortality, categorizing the patient as high-risk (≥ 40%). This single output can trigger a predefined “high-risk” pathway: immediate nephrology consultation, strict urine-output and creatinine surveillance, a zero-balance fluid protocol, and a structured family meeting to discuss goals of care. If the SII trajectory continues to rise beyond 48 h, the pathway escalates to consider immunomodulatory or cytokine-adsorption trials and early renal-replacement therapy. By contrast, patients with identical physiological scores but modest SII values fall into intermediate (15%–40%) or low (< 15%) tiers, warranting progressively less intensive bundles. Thus, the tool converts routine laboratory data into an actionable, precision-medicine decision aid rather than a purely prognostic score.

From an implementation perspective, the nomogram is straightforward to use and relies on data readily available within 24 h of ICU admission. It yields an absolute risk prediction for 1-year survival, which could be helpful for clinicians when communicating prognoses to patients' families or when stratifying patients for clinical trials (e.g., identifying a high-risk subgroup). The incorporation of SII_min and SII_max essentially brings the patient's immunological response into the prognostic model, thereby personalizing the risk assessment beyond generic severity scores. This approach aligns with the move toward precision medicine in critical care—recognizing that two patients with the same APS III score might have different outcomes if one has an exuberant inflammatory response while the other does not [24].

Toward SII-guided interventions does not prove that reacting to SII improves outcomes. Nevertheless, the biomarker lends itself to testable interventional hypotheses. Renal protection: A rapidly rising SII_slope could trigger an automated alert to initiate an AKI prevention bundle (strict fluid targets, avoidance of nephrotoxic antibiotics, early nephrology consult). Immunomodulation: Patients whose SII remains > 90th percentile despite standard care might be candidates for exploratory trials of corticosteroid “pulse” therapy or cytokine adsorption. Resource allocation: Real-time SII tracking could help triage limited CRRT capacity toward those with both biochemical evidence of inflammation and high mortality risk. If such interventional trials confirm clinical benefit, the nomogram could evolve from a purely prognostic device into a precision trigger for immunoinflammatory management—illustrating how early host-response profiling can move critical-care practice beyond “one-size-fits-all” resuscitation.

### 4.1. Limitations

Several limitations of our study should be acknowledged. First, the analysis is based on MIMIC-IV, a single-center, retrospective database. Patient mix, staffing ratios, and treatment protocols in this tertiary academic ICU may differ substantially from community or non-US ICUs, limiting generalizability. To address this, we have initiated a two-step external-validation plan for the future: (i) a temporal validation within MIMIC-IV itself (2008–2012 as a derivation cohort, 2013–2019 as a hold-out test cohort) and (ii) a geographical validation using the multicenter eICU-CRD dataset and a prospective cohort at our institution. Second, although we adjusted for a broad set of demographic and physiologic covariates, several clinically important confounders—such as detailed hemodynamic targets, fluid-balance strategies, nephrotoxic drug exposure, and immunomodulatory therapies—were incompletely captured or entirely absent in the source tables. Future work will incorporate medication administration records, high-resolution waveform data, and causal-inference techniques (e.g., doubly robust propensity weighting) to mitigate unmeasured-treatment bias. Additionally, we examined SII dynamics only within the first 24 h of ICU admission; longer trajectories (48 h or 72 h) might capture biphasic inflammatory patterns and could further enhance risk prediction. Some patients had only one SII measurement on day 1 and were therefore excluded from slope calculations. Finally, although the nomogram was internally bootstrap validated, we have not yet tested it in an external dataset; its discrimination and calibration may attenuate in new settings. A prospective, multi-center study is under development to determine whether SII-guided risk stratification can meaningfully improve clinical decision-making and patient outcomes.

## 5. Conclusions

In critically ill patients, dynamic immune–inflammation indices, including the 24-h SII_slope and the first-day SII_min and SII_max, provide important prognostic information. A steeper rise in SII is associated with higher risk of AKI and mortality, while extreme values of SII help identify patients at risk for poor outcomes. Integrating these biomarkers with traditional severity scores enables more accurate and personalized risk prediction, as demonstrated by our high-performance nomogram. These findings underscore the value of monitoring the immune-inflammatory trajectory in the ICU. The SII-based nomogram could serve as a practical tool for clinicians to stratify risk early in the ICU course and to guide clinical decisions or discussions about prognosis. Future research should focus on external validation of this model and on investigating therapeutic strategies for patients identified as high-risk by dynamic SII changes. By bridging the gap between immunological data and clinical risk assessment, we move closer to a more nuanced management of critically ill patients that accounts for their inflammatory state in real time.

## Figures and Tables

**Table 1:** Baseline characteristics.

Variable	Overall	Non-AKI	AKI	*p*
*n*	17,491	12,906	4585	
Age	65.21 (15.36)	62.17 (16.90)	66.29 (14.63)	< 0.001
Gender = male (%)	10,672 (61.0)	2660 (58.0)	8012 (62.1)	< 0.001
Weight	83.18 (22.97)	76.68 (19.70)	85.47 (23.60)	< 0.001
Height	170.16 (10.49)	169.44 (10.61)	170.33 (10.45)	< 0.001
BMI	30.01 (55.46)	27.09 (6.11)	30.74 (61.90)	0.004
SOFA	5.60 (3.62)	3.93 (2.77)	6.19 (3.70)	< 0.001
APSIII	46.23 (21.98)	37.66 (16.87)	49.27 (22.77)	< 0.001
SAPSII	38.46 (14.47)	32.07 (12.45)	40.73 (14.45)	< 0.001

*Interventions (Boolean for 1st 24 h)*				
Mechanical ventilation use (yes) (%)	45.9	26.8	52.7	< 0.001
Vasopressor use (yes) (%)	43.9	29.6	49.0	< 0.001
Sedative use (yes) (%)	47.4	33.5	52.3	< 0.001

*Comorbidities (Boolean)*				
HF (yes) (%)	31.5	22.1	34.8	< 0.001
AFIB (yes) (%)	1.2	0.9	11.3	0.029
Renal (yes) (%)	23.9	15.6	26.9	< 0.001
Liver (yes) (%)	4.7	3.9	5.0	0.003
COPD (yes) (%)	13.3	11.6	13.9	< 0.001
CAD (yes) (%)	41.4	35.3	43.5	< 0.001
Stroke (yes) (%)	9.5	7.2	10.3	< 0.001
Malignancy (yes) (%)	18.4	17.7	18.6	0.156

*Vital signs (1st 24 h)*				
MAP	82.83 (18.07)	84.85 (17.58)	82.11 (18.19)	< 0.001
Heart_rate	89.00 (20.23)	88.06 (19.77)	89.34 (20.39)	< 0.001
Temperature	36.65 (0.83)	36.69 (0.73)	36.63 (0.87)	< 0.001
Resp_rate	19.63 (6.39)	19.22 (6.11)	19.78 (6.48)	< 0.001

*Laboratory tests (1st 24 h)*				
Na	137.15 (5.37)	137.38 (5.27)	137.06 (5.41)	0.001
K	4.35 (0.84)	4.27 (0.90)	4.38 (0.82)	< 0.001
HCO3	22.03 (4.54)	22.30 (4.18)	21.94 (4.66)	< 0.001
Cl	103.42 (7.00)	103.71 (6.26)	103.32 (7.24)	0.001
Bun	26.70 (22.82)	22.07 (19.75)	28.34 (23.60)	< 0.001
Lactate	2.44 (2.14)	2.13 (1.72)	2.53 (2.25)	< 0.001
Creatinine	1.48 (1.59)	1.11 (1.03)	1.61 (1.73)	< 0.001
PH	7.36 (0.09)	7.37 (0.08)	7.36 (0.10)	< 0.001
PO2	206.06 (129.25)	222.44 (133.77)	202.25 (127.88)	< 0.001
PCO2	43.56 (12.69)	42.55 (11.67)	43.79 (12.89)	< 0.001
BNP (yes) (%)	5.6	4.1	6.1	< 0.001
Troponin (yes) (%)	29.2	23.9	31.1	< 0.001
CK (yes) (%)	19.5	16.1	20.6	< 0.001
Wbc_first	13.59 (12.81)	12.73 (15.03)	13.90 (11.90)	< 0.001
Wbc_min	11.72 (9.35)	11.08 (9.93)	11.94 (9.12)	< 0.001
Wbc_max	15.84 (14.00)	14.27 (16.25)	16.40 (13.07)	< 0.001
Neu_first	10.61 (7.51)	9.49 (6.72)	11.01 (7.73)	< 0.001
Neu_min	10.43 (7.32)	9.36 (6.55)	10.81 (7.54)	< 0.001
Neu_max	10.79 (7.69)	9.58 (6.81)	11.22 (7.93)	< 0.001
Lym_first	1.72 (6.47)	1.79 (6.31)	1.69 (6.53)	0.385
Lym_min	1.65 (5.72)	1.75 (6.20)	1.62 (5.54)	0.187
Lym_max	1.76 (6.71)	1.84 (6.66)	1.73 (6.72)	0.367
Platelet_first	185.76 (103.74)	189.93 (102.02)	184.28 (104.30)	0.002
Platelet_min	169.85 (97.23)	178.69 (96.93)	166.71 (97.14)	< 0.001
Platelet_max	200.20 (102.74)	201.46 (100.35)	199.75 (103.57)	0.332
SII_min	2319.45 (4313.88)	2066.52 (3379.64)	2409.31 (4597.10)	< 0.001
SII_max	2608.85 (4617.29)	2247.42 (3660.04)	2737.25 (4906.39)	< 0.001
SII_slope_24 h	12.06 (57.05)	7.54 (38.75)	13.66 (62.19)	< 0.001

*Note:* Values are presented as mean (standard deviation) for continuous variables and number (percentage) for categorical variables.

## Data Availability

The datasets presented in the current study are available in the MIMIC IV database (https://physionet.org/content/mimiciv/3.0/). The data used and analyzed during the current study are available from the corresponding author (T.Y.) on reasonable request.

## Supporting Information

Supporting Information 1 Supporting Table 1: Baseline characteristics by SII measurement frequency. Values are presented as mean (standard deviation) for continuous variables and number (percentage) for categorical variables.

Supporting Information 2 Supporting Figure 1. Kaplan–Meier survival curves by first-day SII_min quartiles (0–365 days). One-year survival is stratified by quartiles of the minimum systemic immune–inflammation index (SII_min) observed within 24 h of ICU admission: Q1 (lowest) through Q4 (highest). The number at risk at specified time points is shown below the plot. Patients in Q2 demonstrated the highest survival probability (log-rank *p* = 0.047).

Supporting Information 3 Supporting Figure 2. Kaplan–Meier survival curves by first-day SII_max quartiles (0–365 days). One-year survival stratified by quartiles of the maximum SII (SII_max) within the first 24 h. The table of numbers at risk is shown beneath the curves. No statistically significant difference in survival was observed across SII_max quartiles (log-rank *p* = 0.31).

Supporting Information 4 Supporting Figure 3. Kaplan–Meier survival curves by 24-h SII_slope quartiles (0–90 days). Short-term (0–90 days) survival is stratified by quartiles of the linear 24-h SII_slope (SII_slope), representing the rate of change in SII during the first ICU day: Q1 (most negative/sloping downward) to Q4 (most positive/sloping upward). The number at risk at 15-day intervals is shown below. A steeper positive SII_slope is associated with significantly worse survival (log-rank *p* < 0.0001).
