# Supplementary material for: Early 24-Hour Changes in Systemic Immune–Inflammation Index Predict Acute Kidney Injury and Mortality in ICU Patients
Source: Emerg Med Int. 2025 Aug 19;2025:4949299. doi: 10.1155/emmi/4949299 (PMC12380515; doi:10.1155/emmi/4949299)
Supplement: Supporting Information 1 — Supporting Table 1: Baseline characteristics by SII measurement frequency. Values are presented as mean (standard deviation) for continuous variables and number (percentage) for categorical variables. [file 4949299.f1.docx]

# Supplementary Table 1: Baseline Characteristics by SII Measurement Frequency

| Variable | Multiple measurements | Single measurement | *P* |
| --- | --- | --- | --- |
| n | 17491 | 3436 |  |
| age (mean (SD)) | 65.21 (15.36) | 65.35 (17.36) | 0.544 |
| gender = M (%) | 10672 (61.0) | 1837 (53.5) | <0.001 |
| BMI (mean (SD)) | 30.01 (55.46) | 28.84 (8.42) | 0.393 |
| SOFA (mean (SD)) | 5.60 (3.62) | 4.08 (3.17) | <0.001 |
| APSIII (mean (SD)) | 46.23 (21.98) | 42.85 (19.23) | <0.001 |
| Mechanical ventilation use (YES) (%) | 45.9 | 18.9 | <0.001 |
| Vasopressor use (YES) (%) | 43.9 | 18.1 | <0.001 |
| Sedative use (YES) (%) | 47.4 | 18.9 | <0.001 |
| HF (YES) (%) | 31.5 | 30.9 | 0.407 |
| AFIB (YES) (%) | 1.2 | 1.0 | 0.279 |
| Renal (YES) (%) | 23.9 | 24.6 | 0.289 |
| Liver (YES) (%) | 4.7 | 3.0 | <0.001 |
| COPD (YES) (%) | 13.3 | 15.8 | <0.001 |
| CAD (YES) (%) | 41.4 | 23.9 | <0.001 |
| Stroke (YES) (%) | 9.5 | 14.3 | <0.001 |
| Malignancy (YES) (%) | 18.4 | 24.1 | <0.001 |
| SII_min (mean (SD)) | 2172.63 (4147.44) | 2920.02 (4891.64) | <0.001 |
| SII_max (mean (SD)) | 2532.37 (4542.86) | 2921.68 (4898.30) | <0.001 |

Values are presented as mean (standard deviation) for continuous variables and number (percentage) for categorical variables.
