# Supplementary figures and images for: Early 24-Hour Changes in Systemic Immune–Inflammation Index Predict Acute Kidney Injury and Mortality in ICU Patients
Source: Emerg Med Int. 2025 Aug 19;2025:4949299. doi: 10.1155/emmi/4949299 (PMC12380515; doi:10.1155/emmi/4949299)

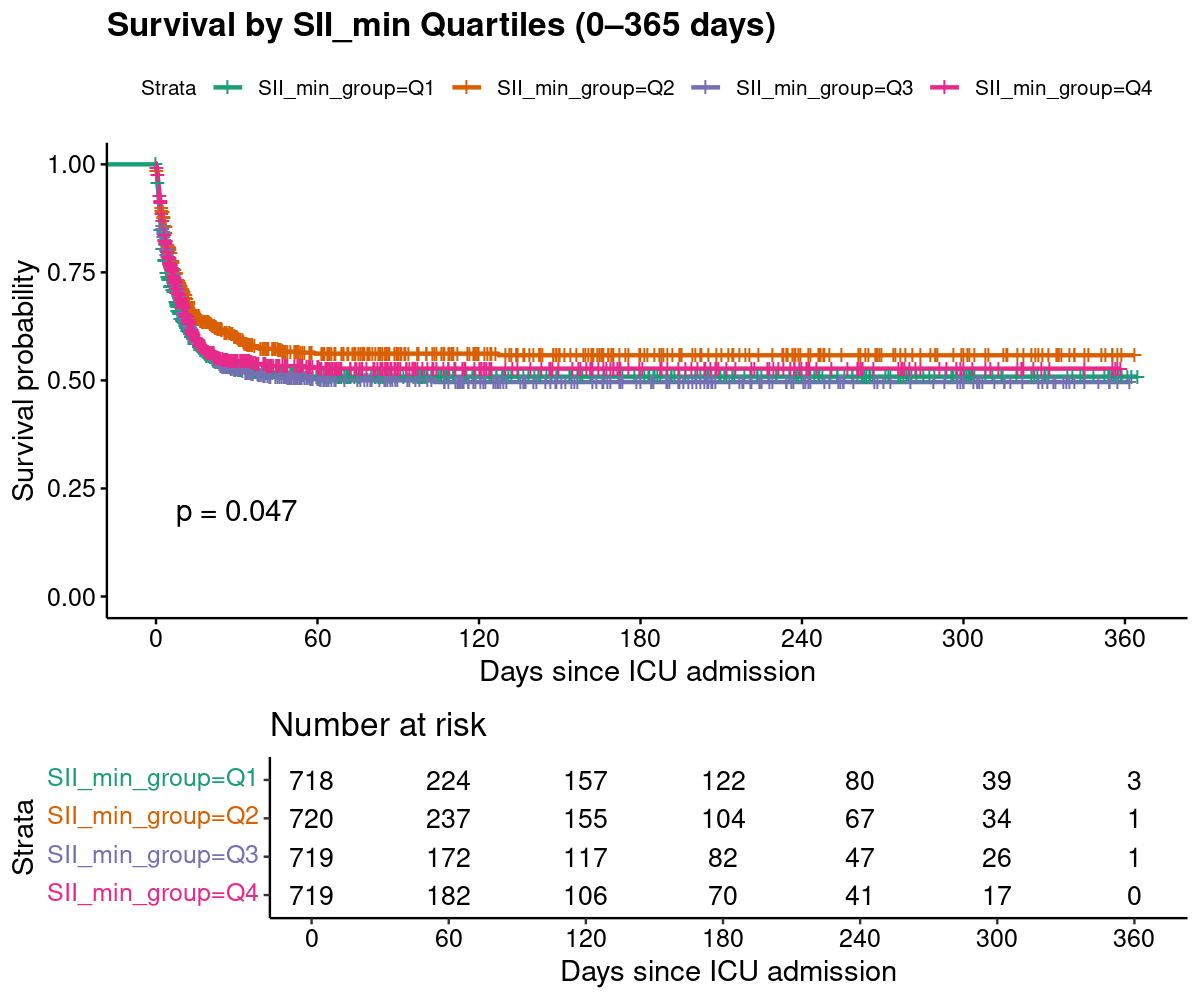

Supplement: Supporting Information 2 — Supporting Figure 1. Kaplan–Meier survival curves by first-day SII_min quartiles (0–365 days). One-year survival is stratified by quartiles of the minimum systemic immune–inflammation index (SII_min) observed within 24 h of ICU admission: Q1 (lowest) through Q4 (highest). The number at risk at specified time points is shown below the plot. Patients in Q2 demonstrated the highest survival probability (log-rank p = 0.047). [file 4949299.f2.png]

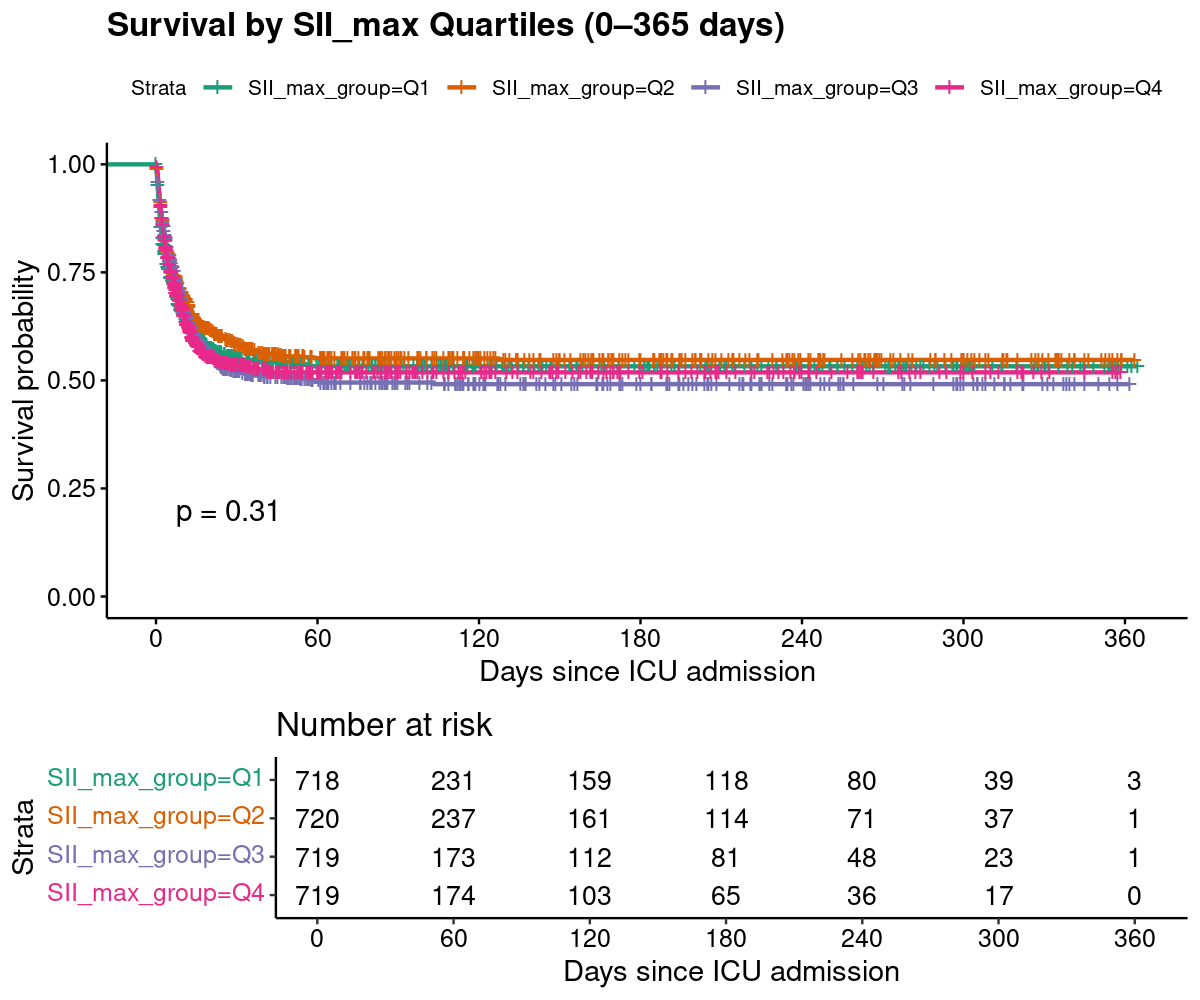

Supplement: Supporting Information 3 — Supporting Figure 2. Kaplan–Meier survival curves by first-day SII_max quartiles (0–365 days). One-year survival stratified by quartiles of the maximum SII (SII_max) within the first 24 h. The table of numbers at risk is shown beneath the curves. No statistically significant difference in survival was observed across SII_max quartiles (log-rank p = 0.31). [file 4949299.f3.png]

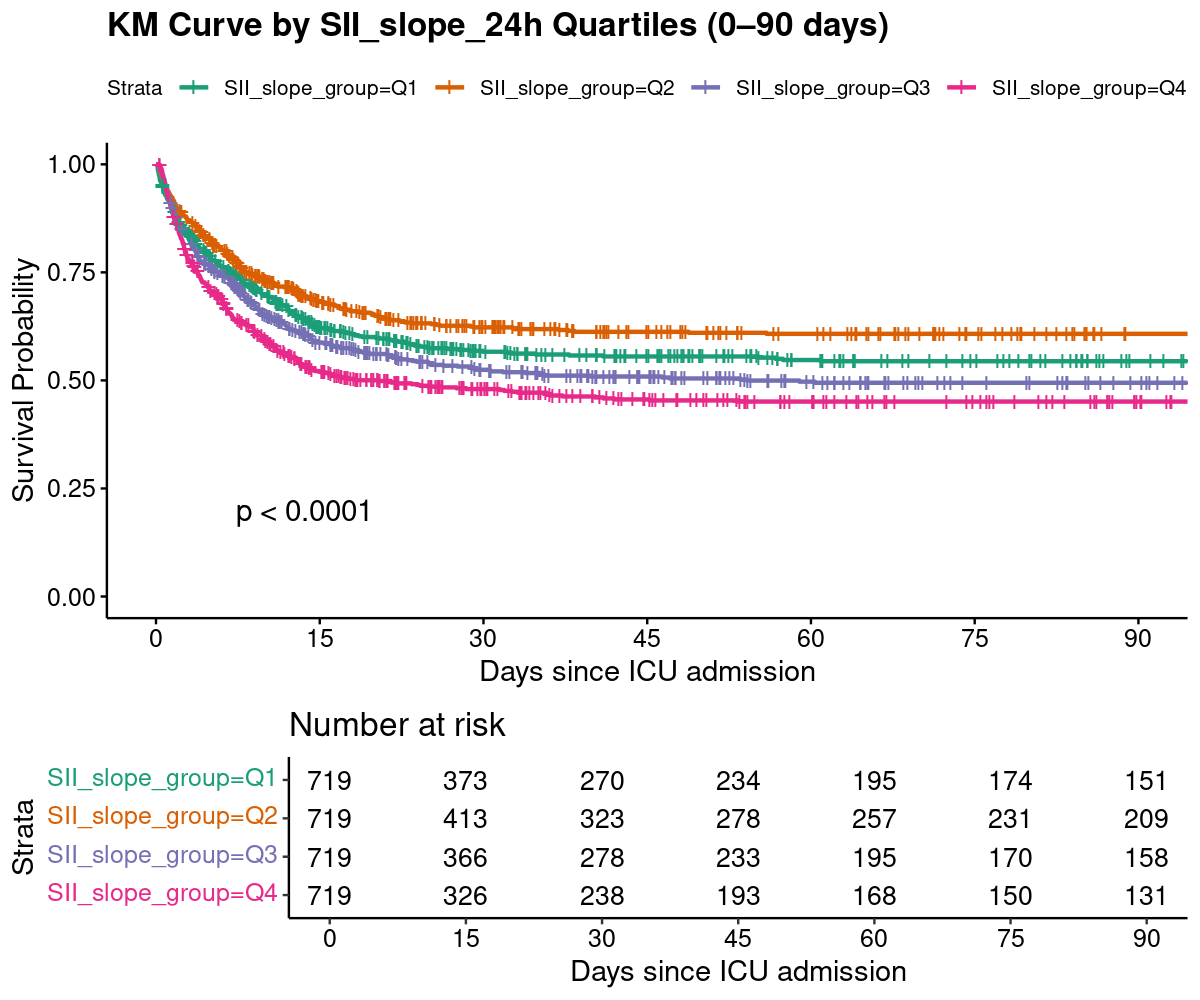

Supplement: Supporting Information 4 — Supporting Figure 3. Kaplan–Meier survival curves by 24-h SII_slope quartiles (0–90 days). Short-term (0–90 days) survival is stratified by quartiles of the linear 24-h SII_slope (SII_slope), representing the rate of change in SII during the first ICU day: Q1 (most negative/sloping downward) to Q4 (most positive/sloping upward). The number at risk at 15-day intervals is shown below. A steeper positive SII_slope is associated with significantly worse survival (log-rank p < 0.0001). [file 4949299.f4.png]
